# Practical Challenges for the Implementation of AI-Based Image Analysis in Ophthalmology Research: Insights and Recommendations from a Swiss Multicentric Study

**DOI:** 10.1055/a-2802-7192

**Published:** 2026-04-20

**Authors:** Tahm Spitznagel, Justus G. Garweg, Chiara Eandi, Gabriela Grimaldi, Moreno Menghini, Aude Ambresin, Sandrine Zweifel, Gabor Mark Somfai

**Affiliations:** 1 Department of Ophthalmology, Stadtspital Zürich Triemli, Zurich, Switzerland; 2 Ophthalmology, Werner H. Spross-Stiftung, Zurich, Switzerland; 3 Clinic for Vitreoretinal Diseases, Berne Eye Clinic, Switzerland; 4 Ophthalmology, Inselspital University Hospital Bern, Switzerland; 5 Rétine médicale, Hôpital ophtalmique Jules-Gonin, Lausanne, Switzerland; 6 Department of Surgical Sciences, University of Torino, Italy; 7 Ophthalmology, Ente Ospedaliero Cantonale, Lugano, Switzerland; 8 Ophthalmology, Swiss Visio Retina Research Center, Lausanne, Switzerland; 9 Ophthalmology, University Hospital of Zürich, Switzerland; 10 Department of Ophthalmology, Semmelweis University, Budapest, Hungary

**Keywords:** RetinAI, Guideline, Retina, image segmentation, multicenter research, artificial intelligence, RetinAI, Bildsegmentierung, multizentrische Forschung, künstliche Intelligenz, Guideline, Netzhaut

## Abstract

**Purpose** To share first experience from the Swiss RetinAI Consortium in applying artificial intelligence (AI)-based optical coherence tomography (OCT) analysis across multiple centres, to highlight practical barriers encountered during implementation, and to outline practical recommendations for future collaborative multicentre AI-based OCT research.

**Methods** The Swiss RetinAI Consortium, consisting of six ophthalmology centres, implemented the FDA-cleared Discovery platform (RetinAI, Bern, Switzerland) for collaborative OCT analysis. Challenges encountered during protocol development for OCT export, anonymisation, data upload, AI-based analysis, and data sharing were systematically collected from all sites involved. These included regulatory, technical, and methodological aspects. The findings were discussed at expert meetings and consolidated into shared guidance for data acquisition and processing. The aim was to provide practical recommendations to support standardised workflows in future multicentre AI-driven research.

**Identified Challenges** Key regulatory, technical, and methodological challenges were identified during multicentre implementation. These included the legal requirement for Swiss-based server hosting, incomplete anonymisation due to heterogeneous export protocols, and non-standardised OCT protocols treated as being equivalent, despite covering different retinal areas. Additional barriers were inter-device variability, ETDRS grid misalignment without the option for manual correction, major segmentation errors requiring extensive review, and unfiltered data exports that often exceeded one million entries, thereby increasing the risk of readout errors. Moreover, the absence of automated quality screening and the lack of cross-centre reproducibility data were identified as important methodological gaps. Together, these challenges shaped our practical recommendations for reliable multicentre AI-based OCT analysis.

**Practical Recommendations** Based on the identified challenges, we derive practical recommendations focusing on standardised anonymised data export procedures, harmonised OCT acquisition protocols, mandatory quality control steps for grid placement and segmentation, and structured strategies for handling large-scale output data. Our recommendations offer a practical roadmap to support future collaborative multicentre AI-based OCT research.

## Zusammenfassung

**Ziel** Ziel dieser Arbeit ist es, erste Erfahrungen des Swiss RetinAI Consortiums bei der multizentrischen Anwendung einer KI-basierten Analyse der optischen Kohärenztomografie (OCT) darzustellen, praxisrelevante Hürden während der Implementierung zu identifizieren und konkrete Empfehlungen für zukünftige multizentrische KI-basierte OCT-Forschungsprojekte abzuleiten.

**Methoden** Das Swiss RetinAI Consortium, bestehend aus 6 ophthalmologischen Zentren, führte im Rahmen eines multizentrischen Implementierungsprozesses eine KI-basierte OCT-Analyse mit der FDA-zugelassenen Discovery-Plattform (RetinAI, Bern, CH) durch. Herausforderungen entlang des OCT-Exports, der Anonymisierung, des Daten-Uploads, der KI-basierten Analyse und des Datenaustauschs wurden multizentrisch systematisch erhoben. Diese umfassten regulatorische, technische und methodische Aspekte. Die Ergebnisse wurden in Expertenrunden diskutiert und in gemeinsame Handlungsempfehlungen überführt.

**Identifizierte Herausforderungen** Zu den identifizierten Herausforderungen zählten die Anforderung an den Serverstandort in der Schweiz, unvollständige Anonymisierung infolge heterogener Exportprozesse sowie nicht standardisierte OCT-Aufnahmeprotokolle, die trotz unterschiedlicher erfasster Netzhautareale als gleichwertig behandelt wurden. Weitere Hürden waren Inter-Device-Variabilität, eine fehlerhafte automatische Platzierung des ETDRS-Gitters ohne manuelle Korrekturmöglichkeit, ausgeprägte Segmentierungsfehler mit hohem manuellem Korrekturaufwand sowie unstrukturierte Datenexporte mit teils über einer Million Einträgen, die das Risiko von Auswertungsfehlern erhöhen. Darüber hinaus stellten fehlende automatisierte Qualitätskontrollen und begrenzte multizentrische Reproduzierbarkeitsdaten relevante methodische Barrieren dar. Diese Erkenntnisse bildeten die Grundlage für die Ableitung praxisorientierter Empfehlungen.

**Praktische Empfehlungen** Auf Basis der identifizierten Herausforderungen haben wir praxisnahe Empfehlungen formuliert, die sich auf standardisierte und vollständig anonymisierte Datenexporte, harmonisierte OCT-Akquisitionsprotokolle, verpflichtende Qualitätskontrollschritte zur Überprüfung von ETDRS-Gitterplatzierung und (Miss-)Segmentierung sowie strukturierte Strategien zur Verarbeitung großer Datenoutputs konzentrieren. Diese Empfehlungen werden in einer praxisorientierten Roadmap zusammengefasst und sollen zukünftige multizentrische KI-basierte OCT-Forschungsprojekte unterstützen.

## Introduction

Therapeutic options for retinal diseases are on the rise, with multiple novel agents already on the market or currently in phase II or III clinical trials 1 , 2 . A deeper understanding of retinal pathophysiology at the microstructural level is essential to identify biomarkers capable of differentiating and predicting therapeutic responses beyond conventional endpoints such as time to drying and re-treatment intervals 3 . Quantitative assessment of retinal microstructure based on optical coherence tomography (OCT) imaging has traditionally relied on segmentation tools that required considerable manual input 4 . As a result, a large body of work has focused on characterizing OCT-derived retinal biomarkers, whose clinical relevance has been well established in prior literature 3 , 5 . Lately, Artificial Intelligence (AI), especially deep learning (DL)-based systems, have made significant progress in detection and quantification of retinal biomarkers offering unprecedented insights into retinal microstructure on a larger scale 6 , 7 , 8 . Although these AI tools have proven successful in single-center studies, they have rarely been implemented within multicenter studies in Switzerland. Leading medical retina centers in Switzerland are increasingly collaborating across the country to systematically gather clinical data on a wide range of retinal conditions. This joint effort aims to generate robust real-world evidence on treatment protocols and patient outcomes, reflecting everyday clinical practice. We had the opportunity to get access to the RetinAI Discovery platform (Ikerian Inc, Bern, Switzerland). Its AI-based image analysis modules are CE-marked as Class IIa medical devices according to the Medical Device Regulation (EU) 2017/745. In the United States, RetinAI Discovery has received FDA 510(k) clearance (K211715) as an ophthalmic medical image management system, authorizing the storage, archiving and management of ophthalmic images including manual annotation and measurements. However, the automated AI-based image analysis algorithms integrated within the platform are designated for research use only and are not cleared for clinical decision making. This opportunity was made possible through an unrestricted grant from Roche, Switzerland, to found the RetinAI consortium, and we are sharing our experience on how to handle the inherent hurdles and problems during implementation. The Discovery platform offers a clinically certified and widely used image and data management environment for automated AI-based OCT segmentation and biomarker extraction in a research context, with minimal need for human interaction. By pooling expertise, infrastructure, and resources, the RetinAI consortium is establishing a nationwide framework that will not only enhance the understanding of disease management but hopefully also provide valuable insights for optimizing therapeutic strategies and improving patient care throughout the country. In this opinion paper, our primary objective is to share first multicenter implementation experiences, systematically highlight regulatory, technical, and methodological challenges, and formulate practical recommendations for future collaborative AI-based OCT research.

The consortium represented by the authors currently includes six large retinal expert centers from different regions in Switzerland: the University Hospital of Zurich, Stadtspital Zürich, Jules-Gonin Eye Hospital, Ente Ospedaliero Cantonale, Swiss Visio Retina Research Center and Berne Eye Clinic. Together, these centers aim to generate robust evidence on AI-derived OCT biomarkers which could support decision-making in clinical practice and in clinical study settings.

The lessons learned during the collaborative process will hopefully provide practical insights into the technical and methodological aspects of implementation and facilitate access to this technology for future users based on our recommendations.

## Methods

Practical challenges were collected iteratively throughout this process using (i) notes from regular consortium meetings, (ii) email correspondence among the participating clinical and technical teams, and (iii) a shared issue log that captured problems encountered during pilot data uploads, anonymization procedures, and early segmentation analyses. These observations were subsequently consolidated and grouped into thematic domains, which form the structure of the following sections.

### Section I – data protection issues

Under Swiss human research law, servers hosting data from patients treated in Switzerland must be physically located within Swiss territory. Depending on the provider, such infrastructure may need to be newly established and commissioned, which is associated with legal and organizational hurdles and requires approval from ethics and legal committees. Once the data hosting has technically been established, each participating institution must sign a data transfer agreement, which must be reviewed and validated by the institutional legal departments and receive approval by the institutional ethics committee and the data governance board.

Retinal imaging data are unique and categorized as specifically vulnerable data, as they may remain indirectly traceable to individuals even after proper de-identification. Institutional and national data protection guidelines must be strictly followed before any imaging data are uploaded and shared in a cloud environment. Only with a detailed close description of de-identification and data hosting following all formal requirements, an ethics application can be submitted. Comparable regulatory complexity may apply in other countries outside Switzerland.

### Section II – anonymized data export

All centers within our network use Heidelberg Spectralis OCT systems in combination with the Heidelberg Explorer 2 (HEYEX) software. However, the anonymized export function of the HEYEX software did not perform equally well on a technical level across departments. All centers exported OCT using the E2E format, for which two anonymization options were available (“Export to drive (anonymized)” and “Anonymisiert”). In some hospitals, these buttons may appear under slightly different names depending on local HEYEX configuration. As the second option was not preinstalled in all clinics, some sites used the default “Export to drive (anonymized)” function, which resulted in residual metadata that remained technically reconstructable, including patient first and last name, date of birth and the date of image acquisition. Therefore, both de-identification extensions within HEYEX and standard operating procedures for the export of anonymized patient data, including random quality controls, need to be established before the start of imaging data export. These issues relate exclusively to the HEYEX export process prior to upload. However, in cases where residual metadata might persist in the exported files, the platformʼs internal handling of such information is not publicly documented.

### Section III – comparability of OCT scans and OCT devices

**Fig. 1:**
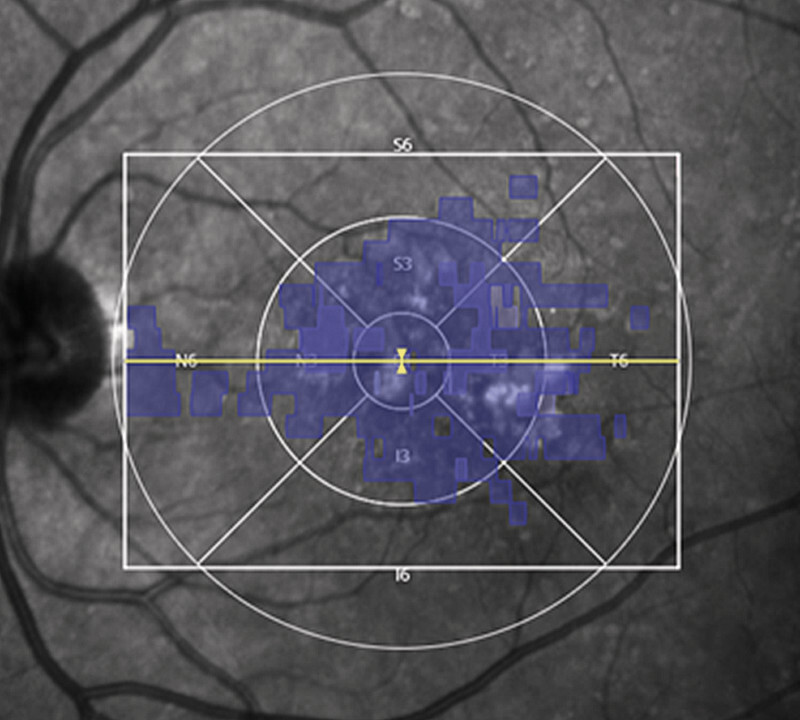
6 × 6 mm ETDRS grid overlay exceeding the scan area, leading to incomplete regional coverage that is not apparent from the analysis output and requires manual verification.

Even when using the same OCT devices, a meaningful cross-center comparison of imaging biomarkers relies on a pre-study agreement of standardized scanning protocols to prevent labor-intensive post-hoc harmonization. In clinical routine, scan protocols commonly differ between centers in both resolution and the covered retinal area ( Fig. 1 ). Images may range from small high-resolution macular scans (3 × 3 mm ^2^ ) to the standard 6 × 6 mm ^2^ protocol used across our network, with some centers occasionally acquiring larger fields of view depending on local routine practice. B-scan densities may vary from 10 to over 100 per volume, depending on patient compliance and fixation stability, with 49 B-scans most often used in our network. Overall, scan density is closely linked to the axial resolution. This may not play a crucial role in clinical routine, but limits comparability and necessitates manual review to extract comparable information from the analysis protocol deposited in the corresponding csv files.

The RetinAI Discovery platform enables OCT segmentation across multiple device manufacturers, such as the Zeiss Cirrus and Topcon Maestro and Triton OCT devices. However, as all centers in our network use Heidelberg Spectralis OCT devices, our data do not allow us to assess the inter-device accuracy if machines from different manufacturers are used.

### Section IV – ETDRS grid placement

**Fig. 2:**
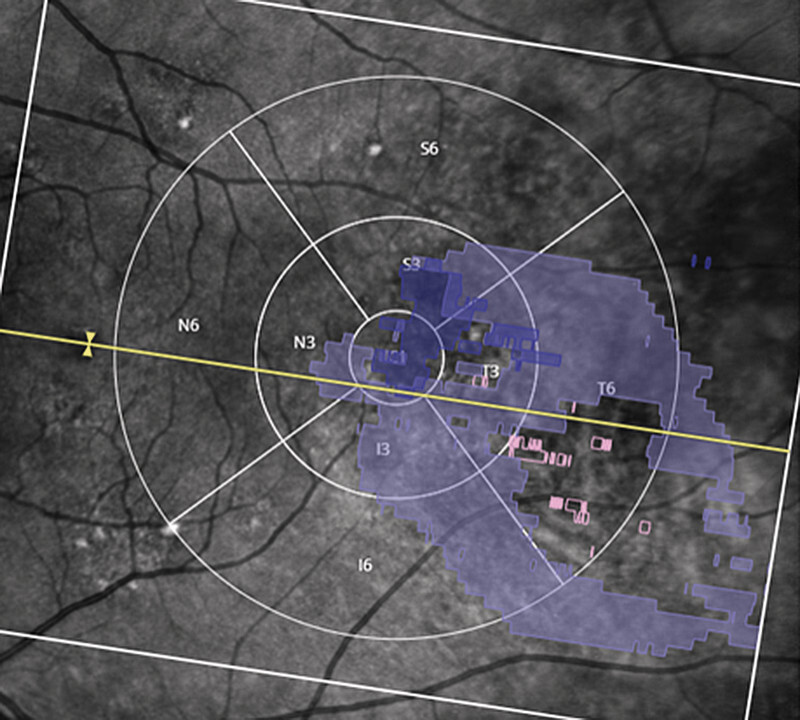
Misplacement of the ETDRS grid with the foveal center located outside the central subfield, leading to incorrect regional assignment that is not apparent from the analysis output and only detectable through manual review. The fovea is marked by the yellow arrow.

Automated ETDRS grid placement can result in misalignment, which cannot be manually corrected or adjusted within the Discovery platform at present ( Fig. 2 ). For this reason, eyes with ETDRS grid displacement currently need to be excluded from further analysis to avoid bias in the results. In our pilot uploads across centers, approximately 7% of OCT volumes (40 out of 600; unpublished data) showed clear grid misalignment requiring exclusion. Misplacement occurred predominantly in eyes with poor visual acuity and fixation problems, where the automated identification of the foveal center failed.

### Section V – segmentation errors

**Fig. 3:**
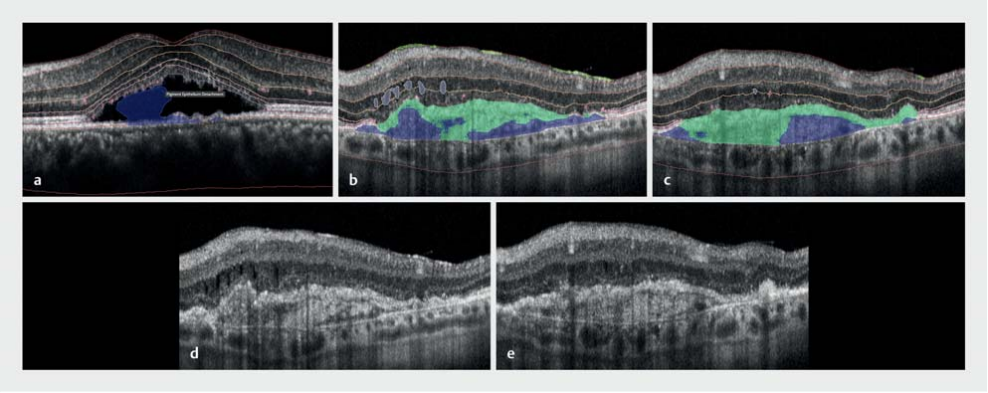
Examples of segmentation errors by the RetinAI Discovery platform. **a** Example of an eye with central serous chorioretinopathy, illustrating misclassification of the subretinal fluid compartment, with approximately 34% of the subretinal fluid segmented incorrectly as pigment epithelial detachment. **b** , **c** Two adjacent B-scans from an eye with nAMD showing inconsistent automated segmentation results. Amorphous material is highlighted in green and PED in blue. Despite their direct spatial adjacency, segmentation labels differ between the two B-scans, indicating limited slice-to-slice consistency (segmentation propagation) of the automated segmentation. **d** , **e** Corresponding raw B-scans to ( **b** , **c** ) without segmentation overlay, demonstrating comparable underlying retinal morphology.

Manual correction of segmentation errors is possible but is highly labor-intensive. Corrections must be manually performed for each B-scan and each OCT volume individually, as the current version of the platform does not support segmentation propagation across adjacent B-scans, resulting in a significant workload, particularly for larger longitudinal datasets. Fig. 3 shows an example of incorrect segmentation by the RetinAI Discovery platform.

### Section VI – Data volume and analytical complexity

The volume of data exported by the Discovery platform is huge, delivering. csv files with several hundred thousand to up to over a million data points per export. While a basic filter by upload date is available, users cannot restrict export to specific biomarkers or data fields. As a result, all available parameters are exported by default, which increases analytic complexity and raises the risk of readout errors, not because of large clinical datasets, but because of the large number of exported outcome variables and parameters per OCT volume. This may be overcome by the development and use of selective data extraction algorithms, which again harbors the risk of data structure errors if individual imaging exports are incomplete or inconsistent.

### Section VII – reproducibility of results

Although RetinAI Discovery has obtained regulatory approval for its intended use, no published studies have systematically evaluated the reproducibility of its automated layer or fluid segmentation outputs in clinical datasets. Based on our experience, a reproducibility study – ideally comparing different scan protocols using the same OCT device – is an unmet need but would be essential to exclude any relevant algorithmic errors.

### Section VIII – clinical adoption and future outlook

The RetinAI Discovery platform is currently intended for research use only and not for diagnostic or therapeutic decision-making. Accordingly, the following section discusses clinical adoption in a forward-looking sense, focusing on potential future integration rather than current clinical use.

Despite significant progress in AI-based retinal image analysis, clinical adoption remains limited. In everyday practice, most clinicians, including those within our consortium, do not rely on AI tools for diagnostic or therapeutic decision-making in clinical routine. This gap highlights a critical distinction between research feasibility and real-world applicability 9 , 10 , 11 , 12 . This problem has been identified by RetinAI, and the upcoming release of an improved automated segmentation algorithm has been announced by the company.

While AI tools demonstrate strong potential in research environments, their current use in routine clinical care is hindered by the need for manual review of segmentation outputs, while a seamless automated integration of imaging data without confirmation of segmentation accuracy into electronic health records is another critical point in clinical practice, possibly leading to wrong clinical decisions. The lack of regulatory frameworks that define the translation of AI outputs into actionable clinical decisions is another unmet need. Until these barriers are overcome, AI will remain a supplementary research instrument rather than a trusted clinical tool.

Nevertheless, the rapid evolution of AI models, increasing interest from industry partners, and the emergence of multicenter collaborations suggest a trajectory toward more practical and reliable tools. Future developments will need to focus on validated reproducibility, vendor-independent compatibility, and the identification of clinically meaningful biomarkers that directly influence patient management.

Bridging the gap between technical capability and clinical usability will require not only algorithmic robustness but also regulatory alignment, workflow integration, and formal assessment of AI impact on clinical outcomes.

Based on the challenges identified, we have developed some practical recommendations for multicenter AI-based OCT research in Table 1 , providing a practical roadmap for implementation.

**Table:** **Table 1** Practical roadmap for multicenter implementation of AI-based OCT analysis.

Step	Recommendation	Rationale
1	Establish standardized workflows for fully anonymized and metadata-clean OCT data export	Ensure data protection compliance and prevent residual metadata
2	Implement harmonized OCT acquisition protocols across centers	Improve cross-center comparability and reduce post-hoc harmonization
3	Perform mandatory manual verification of ETDRS grid placement	Identify and exclude cases with grid misalignment
4	Conduct targeted manual review of major segmentation errors, particularly in complex pathology	Prevent systematic misclassification of retinal compartments
5	Apply dedicated data extraction and filtering algorithms for large-scale output files	Reduce readout errors and analytic complexity in high-dimensional datasets

This opinion paper demonstrates that the successful implementation of AI-based OCT analysis in multicenter research settings is primarily constrained by regulatory, technical, and methodological challenges rather than by algorithmic performance alone. Based on our multicenter experience, key obstacles include data anonymization procedures, heterogeneous acquisition protocols, automated grid placement, segmentation reliability, and the management and interpretation of large-scale output data.

Addressing these challenges through standardized workflows, clearly defined quality control measures, and harmonized standard operating procedures for data acquisition and processing is the fundament for achieving reliable and reproducible AI-based analysis outcomes. By systematically accounting for these practical considerations already during the conception phase, AI tools may generate clinically meaningful insights in multicenter real-world studies.
